# Supplementary material for: Listeria monocytogenes Biofilms Are Planktonic Cell Factories despite Peracetic Acid Exposure under Continuous Flow Conditions
Source: Antibiotics (Basel). 2023 Jan 19;12(2):209. doi: 10.3390/antibiotics12020209 (PMC9952409; doi:10.3390/antibiotics12020209)
Supplement: Supplementary file 1 [file antibiotics-12-00209-s001.zip › antibiotics-2130486-supplementary.pdf]

Control Experiments

1. Effect of Sterile Distilled Water (Diluent For PAA) on Sessile *L. monocytogenes* serovar 1/2a EGD-e Community Cultivated at 10 °C, 22 °C and 37 °C

The influence of the diluent (dH_2O) used to constitute the working stocks of various concentrations of PAA on mature *L. monocytogenes* serovar 1/2a EGD-e biofilms cultivated at 10 °C, 22 °C and 37 °C was evaluated. When sterile dH_2O was dosed for 20 minutes into the system very similar responses were observed irrespective of cultivation temperature. Briefly, the metabolic activity of all the biofilms were affected by the introduction of the water, with a decrease in activity observed. This reduction in activity was, however, brief and in general less than 5 h in duration before a recovery in activity resumed (Figure S1B, D, and F, black line). The biofilm biomass showed a similar trend across the temperature range tested, with an initial spike in absorbance followed by a decrease and a recovery (Supplementary Figure 1B, D and F, red line). After recovery, the biofilm biomass across all the temperatures stabilized at increased values, nearly double that of the pre-dosing steady-state. The biofilm-derived planktonic cells exhibited remarkable stability across the experimental period, which included biofilm cultivation, dosing, and recovery, with cell yields during the establishment of the biofilm and post-treatment differing less than 1 log CFU.ml^{-1} (Figure S1A, C and E). The exposure to sterile dH_2O generally had minimal effect on cell yields as can be seen by the numbers 1 h after treatment (hatched bars, Figure S1A, C and E). Across all the temperatures the biofilms produced cells at a relatively consistent rate.

2. Abiotic Controls for In-Situ Biofilm Biomass and Metabolic Activity Monitoring Under Continuous-Flow Conditions

The influence of the oxidizing PAA sanitizer on the uninoculated CEMS-BioSpec system at the various temperatures was examined to account for any contribution to CO_2 evolution or absorbance measurements that can be attributed to abiotic factors. The introduction of all the PAA concentrations caused a spike in CO_2 detected. However, these increases were small relative to biotic responses and was promptly followed by a decrease in concentration across the temperature ranges (Figure S2). The PAA concentrations had minimal effect on the absorbance values measured by the system; any changes were significantly smaller than biotic responses.

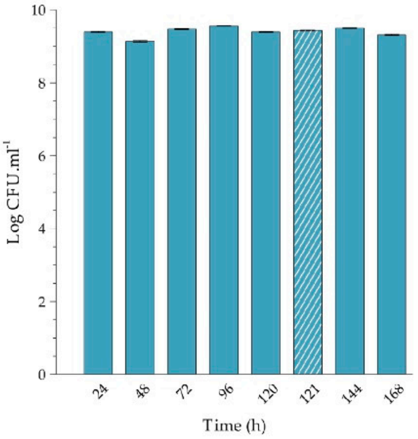
Log CFU.ml-1
Time (h)
24 48 72 96 120 121 144 168
0 2 4 6 8 10

(**a**)

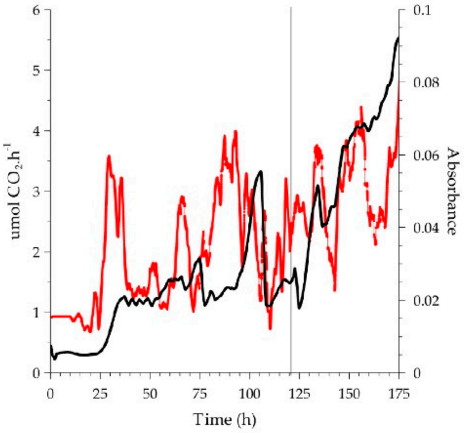
umol CO2.h-1
Absorbance
Time (h)
0 25 50 75 100 125 150 175

(**b**)

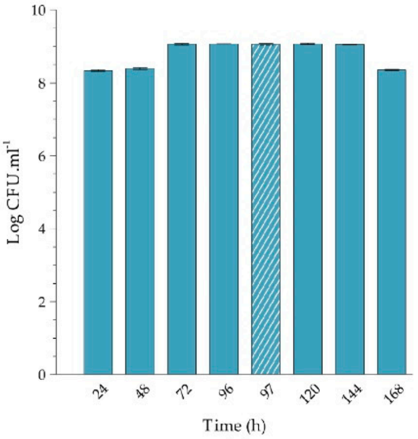
Log CFU.ml-1
Time (h)
24 48 72 96 97 120 144 168
0 2 4 6 8 10

(**c**)

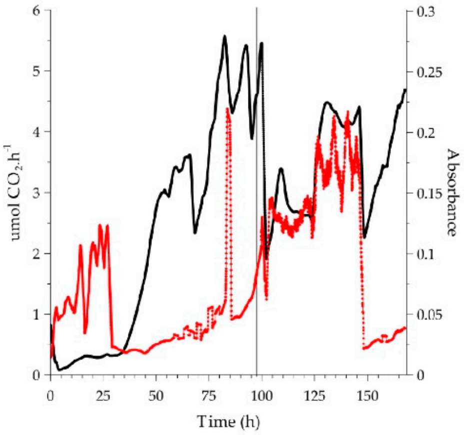
umol CO2.h-1
Absorbance
Time (h)
0 25 50 75 100 125 150

(**d**)

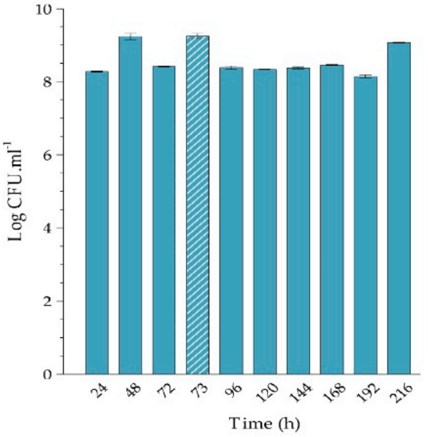
Log CFU.ml-1
Time (h)
24 48 72 73 96 120 144 168 192 216
0 2 4 6 8 10

(**e**)

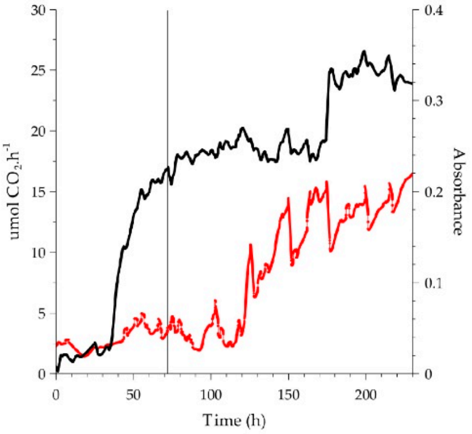
umol CO2.h-1
Absorbance
Time (h)
0 50 100 150 200

(**f**)

Figure S1. Sessile growth of *Listeria monocytogenes* serovar 1/2a EDG-e at (**a**) 10 °C (refrigerated conditions), (**b**) 22 °C (room temperature) and (**c**) 37 °C (human body temperature) exposed to single (slug) dose of sterile dH2O (diluent used for PAA solutions) for 20 minutes dosing periods under continuous flow conditions. The *Listeria* biofilms were cultivated in the combined CEMS-BioSpec system with 10 % BHI. Changes in biofilm metabolic activity (black line) and biofilm biomass (red line) were monitored in real-time with the dosing period indicated by vertical black line at (**b**) 120 h, (**d**) 96 h and (**f**) 72 h. Planktonic cell yields from sessile community cultivated at (**a**) 10 °C before and after dosing with sterile dH2O, (**c**) 22 °C before and after dosing with sterile dH2O and (**e**) 37 °C before and after dosing with sterile dH2O. Cross-hatched bars represent planktonic cell yields 1 h after dosing (10 °C-121 h, 22 °C-97 h and 37 °C-73 h respectively) of the sessile communities. All data are a representation of biological duplicates. Each bar represents the logged mean of triplicate plate counts with the error bar representing the standard deviation.

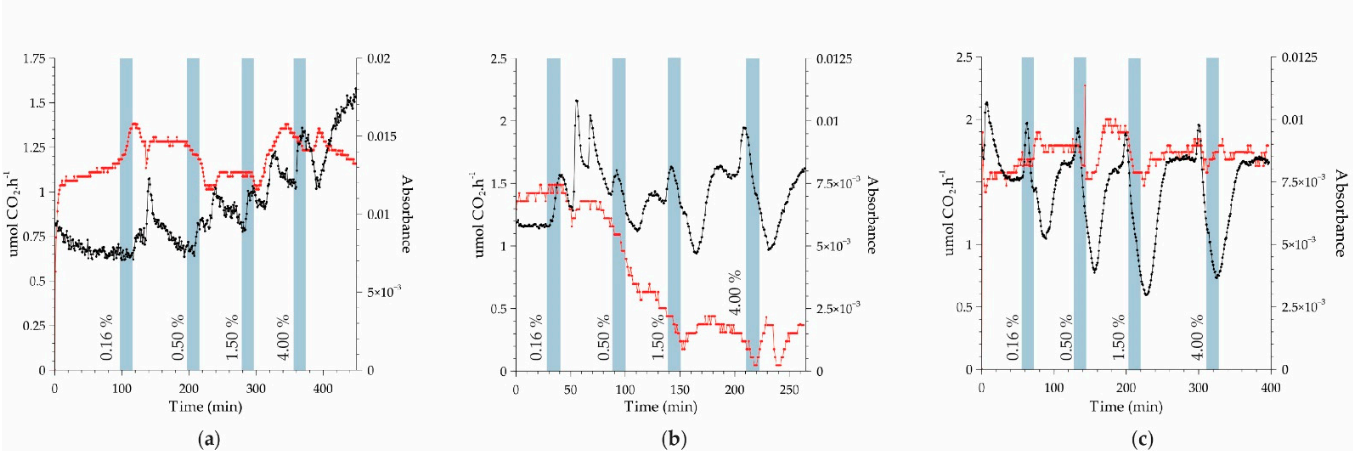

Figure S2. Abiotic influences of increasing concentrations of PAA, diluted in sterile dH2O (0.16 %, 0.5 %, 1.5 % and 4.0 % v/v peracetic acid in sterile dH2O and 10 % m/v BHI) on the uninoculated CEMS-BioSpec system. Abiotic effects of the sequential introduction of increasing concentrations of PAA in dH2O (0.16 %, 0.5 %, 1.5 % and 4.0 % v/v respectively, as indicated by blue shaded regions) with the effects of 10 % BHI before, between doses and after dosing (unshaded regions) on CO2 evolution rates (black line) and absorbance measurements (red line) were assessed for the three different temperatures; (**a**) 10 °C (refrigerated conditions), (**b**) 22 °C (room temperature) and (**c**) 37 °C (human body temperature). All data is representation of triplicate experiments.
